# DO INTERNALIZED AGE STEREOTYPES MEDIATE THE RELATIONSHIP BETWEEN VOLUNTEERING AND PURPOSE IN LIFE FOR ADULTS 50+?

**DOI:** 10.1093/geroni/igad104.2699

**Published:** 2023-12-21

**Authors:** Andrew Steward, Yating Zhu

**Affiliations:** University of Wisconsin-Milwaukee, Milwaukee, Wisconsin, United States; University of Denver, Denver, Colorado, United States

## Abstract

The productive aging literature suggests that volunteering plays a crucial role in enhancing psychosocial well-being among older adults. A growing body of research based in stereotype embodiment theory also demonstrates significant negative health impacts of internalized age stereotypes. Yet, limited research explores which social engagement activities may both reduce internalized ageism and enhance psychosocial health as people age. This cross-sectional study investigated whether internalized age stereotypes mediate the relationship between volunteering and a sense of purpose in life for adults 50+. A convenience sample of volunteers (n = 154) 50+ years of age living in the U.S. Mountain West completed a 15-minute, online survey. The independent variable was number of volunteer hours per week (mean = 6.39, SD = 5.47). The dependent variable was purpose in life measured by six items from the positively worded, five-point purpose in life subscale of the Ryff psychological well-being scale (α = .75; mean = 3.93, SD = 0.61). Drawing from the self-stereotypes of aging scale, the indirect effects of five internalized positive (e.g., “wise” and “capable”) and five negative (e.g., “grumpy” and “helpless”) age stereotypes were tested. Results indicate that increased internalized positive, not negative, age stereotypes partially mediated the relationship between volunteer hours and purpose in life while holding constant age, gender, race, functional limitation, education, employment, self-rated health, and previous volunteer experience. The findings suggest that internalizing positive age stereotypes may function as a form of esteem and contribute to an enhanced sense of purpose in later life.

